# Animal Rights as a Mainstream Phenomenon

**DOI:** 10.3390/ani1010102

**Published:** 2011-01-19

**Authors:** Bernard E. Rollin

**Affiliations:** Department of Philosophy, Colorado State University, Fort Collins, CO 80523, USA; E-Mail: Bernard.rollin@colostate.edu; Tel.: +1-970-491-6885; Fax: +1-970-491-4900

**Keywords:** animal rights, animal husbandry, social ethic, anti-cruelty ethic, recollection

## Abstract

**Simple Summary:**

The twentieth century has witnessed a bewildering array of ethical revolutions, from civil rights to environmentalism to feminism. Often ignored is the rise of massive societal concern across the world regarding animal treatment. Regulation of animal research exists in virtually all western countries, and reform of “factory farming” is regnant in Europe and rapidly emerging in the United States. Opponents of concern for animals often dismiss the phenomenon as rooted in emotion and extremist lack of appreciation of how unrestricted animal use has improved human life. Such a view totally ignores the rational ethical basis for elevating legal protection for animals, as explained in this essay.

**Abstract:**

Businesses and professions must stay in accord with social ethics, or risk losing their autonomy. A major social ethical issue that has emerged in the past four decades is the treatment of animals in various areas of human use. Society's moral concern has outgrown the traditional ethic of animal cruelty that began in biblical times and is encoded in the laws of all civilized societies. There are five major reasons for this new social concern, most importantly, the replacement of husbandry-based agriculture with industrial agriculture. This loss of husbandry to industry has threatened the traditional fair contract between humans and animals, and resulted in significant amounts of animal suffering arising on four different fronts. Because such suffering is not occasioned by cruelty, a new ethic for animals was required to express social concerns. Since ethics proceed from preexisting ethics rather than *ex nihilo*, society has looked to its ethic for humans, appropriately modified, to find moral categories applicable to animals. This concept of legally encoded rights for animals has emerged as a plausible vehicle for reform.

The last 50 years have witnessed a dazzling array of social ethical revolutions in Western society. Such moral movements as feminism, civil rights, environmentalism, affirmative action, consumer advocacy, pro- and anti-abortion activism, homosexual rights, children's rights, the student movement, antiwar activism, public rejection of biotechnology, have forever changed the way governments and public institutions comport themselves. This is equally true for private enterprise: to be successful, businesses must be seen as operating solidly in harmony with changing and emerging social ethics. It is arguable that morally based boycotting of South African business was instrumental in bringing about the end of apartheid, and similar boycotting of some farm products in the U.S. led to significant improvements in the living situations of farm workers. It is de rigeur for major corporations to have reasonable numbers of minorities visibly peopling their ranks, and for liquor companies to advertise on behalf of moderation in alcohol consumption. Cigarette companies now press upon the public a message that cigarettes kill, and extol their involvement in protecting battered women; and forestry and oil companies spend millions (even billions) to persuade the public of their environmental commitments. CNN reported that “green” investment funds grew significantly faster than ordinary funds, and reports of child labor or sweatshop working conditions can literally destroy product markets overnight.

Not only is success tied to accord with social ethics but, even more fundamentally, freedom and autonomy are as well. Every profession—be it medicine, law or agriculture—is given freedom by the social ethic to pursue its aims. In return, society basically says to professions it does not understand well enough to regulate, “you regulate yourselves the way we would regulate you if we understood what you do, which we don't. But we will know if you don't self-regulate properly and then we will regulate you, despite our lack of understanding.” For example, some years ago, Congress became concerned about excessive use of antibiotic in animal feeds, and concluded that veterinarians were a major source of the problem. As a result, Congress was about to ban extra-label drug use by veterinarians, a move that would have killed veterinary medicine as we know it. However, through extensive efforts to educate legislators, such legislation did not proceed to law.

One major social ethical concern that has developed over the last four decades is a significant emphasis on the treatment of animals used by society for various purposes. It is easy to demonstrate the degree to which these concerns have seized the public imagination. According to both the U.S. National Cattlemen's Beef Association and the National Institutes of Health (the latter being the source of funding for the majority of biomedical research in the U.S.), both groups not inclined to exaggerate the influence of animal ethics, by the early 1990s Congress had been consistently receiving more letters, phone calls, faxes, e-mails and personal contacts on animal-related issues than on any other topic [1,2].

Whereas 30 years ago one would have found no bills pending in the U.S. Congress relating to animal welfare, recent years have witnessed dozens of such bills annually, with even more proliferating at the state level, as we shall explain in detail. The federal bills have ranged from attempts to prevent duplication in animal research, to saving marine mammals from becoming victims of tuna fishermen, to preventing importation of ivory, to curtailing the parrot trade. State laws passed in large numbers have increasingly prevented the use of live or dead shelter animals for biomedical research and training and have focused on myriad other areas of animal welfare. Eight states have abolished the steel-jawed leghold trap, as have some 90 countries [3]. When Colorado's politically appointed Wildlife Commission failed to act on a recommendation from the Division of Wildlife to abolish the spring bear hunt (because hunters were liable to shoot lactating mothers, leaving their orphaned cubs to die of starvation), the general public ended the hunt through a popular referendum. Seventy percent of Colorado's population voted for this as a *constitutional amendment* [4]. In Ontario, the environmental minister stopped a similar hunt by executive fiat in response to social ethical concern [5]. California abolished the hunting of mountain lions, and state fishery management agencies have been taking a hard look at catch-and-release programs on humane grounds [6].

In fact, wildlife managers have worried, in academic journals, about “management by referendum” for humane reasons. According to the director of the American Quarter Horse Association, the number of state bills related to horse welfare filled a telephone-book-sized volume in 1998 alone [7]. Public sentiment for equine welfare in California carried a bill through the state legislature making the slaughter of horses or shipping of horses for slaughter a felony in that state and the end of horse slaughter in the U.S. followed. Municipalities have passed ordinances ranging from the abolition of rodeos, circuses, and zoos to the protection of prairie dogs and, in the case of Cambridge, Massachusetts (a biomedical Mecca), the strictest laws in the world regulating research.

Even more dramatic, perhaps, is the worldwide proliferation of laws to protect laboratory animals. In the United States, for example, two major pieces of legislation, which I helped draft and defend before Congress, regulating and constraining the use and treatment of animals in research were passed by the U.S. Congress in 1985, despite vigorous opposition from the powerful biomedical research and medical lobbies. This opposition included well-financed, highly visible advertisements and media promotions indicating that human health and medical progress would be harmed by implementation of such legislation. There was even a less than subtle film titled “Will I Be All Right, Doctor?” the query coming from a sick child, the response coming from a pediatrician who affirmed, in essence, “You will be if ‘they’ leave us alone to do as we wish with animals.” With social concern for laboratory animals unmitigated by such threats, research animal protection laws moved easily through Congress and have been implemented at considerable cost to taxpayers. When I testified before Congress on behalf of this law in 1982, a literature search in the Library of Congress turned up no papers in the scientific literature on laboratory animal analgesia and only two on animal analgesia, one of which said “there ought to be papers.” Now there are over 11,000.

In 1986, Britain superseded its pioneering act of 1876 with new laws aimed at strengthening public confidence in the welfare of experimental animals [8]. Many other countries have moved or are moving in a similar direction, despite the fact that some 90% of laboratory animals are rats and mice, not the most cuddly and lovable of animals. Research on Great Apes has been truncated across the world.

Many animal uses seen as frivolous by the public have been abolished without legislation. Toxicological testing of cosmetics on animals has been truncated; companies such as the Body Shop have been wildly successful internationally by totally disavowing such testing, and free-range egg production is a growth industry across the Western world. Greyhound racing in the U.S. has declined, in part for animal welfare reasons, with the Indiana veterinary community spearheading the effort to prevent greyhound racing from coming into the state. Zoos that are little more than prisons for animals (the state of the art during my youth) have all but disappeared, and the very existence of zoos is being increasingly challenged, despite the public's unabashed love of seeing animals. And, as Gaskell and his associates' work has revealed [9], genetic engineering has been rejected in Europe not, as commonly believed, for reasons of risk but for reasons of ethics; in part for reasons of animal ethics. Similar reasons (*i.e.*, fear of harming cattle) have, in part, driven European rejection of bovine somatotropin (BST). Rodeos such as the Houston Livestock Show have, in essence, banned jerking of calves in roping, despite opposition from the Professional Rodeo Cowboys Association, who themselves never show the actual roping of a calf on national television.

Inevitably, agriculture has felt the force of social concern with animal treatment—indeed, it is arguable that contemporary concern in society with the treatment of farm animals in modern production systems blazed the trail leading to a new ethic for animals. As early as 1965, British society took notice of what the public saw as an alarming tendency to industrialize animal agriculture by chartering the Brambell Commission, a group of scientists under the leadership of Sir Rogers Brambell, who affirmed that any agricultural system failing to meet the needs and natures of animals was morally unacceptable [10]. Though the Brambell Commission recommendations enjoyed no regulatory status, they served as a moral lighthouse for European social thought. In 1988, the Swedish Parliament passed, virtually unopposed, what the *New York Times* call a “Bill of Rights” for farm animals, abolishing in Sweden, in a series of timed steps, the confinement systems currently dominating North American agriculture [11]. Much of northern Europe has followed suit, and the European Union is moving in a similar direction, and sow stalls must be eliminated in by 2011 [12].

Although the U.S. has been a latecomer to agricultural issues, things have moved rapidly, with referenda pressed by HSUS abolishing sow stalls, battery cages, and veal crates across the U.S. My own work attests to this tendency. In 2007, over two days of dialogue, I convinced Smithfield Farms, the world's largest pork producer, to phase out gestation crates. In 2008, the Pew Commission, on which I served as the advocate for farm animal welfare, called for the end of high confinement animal agriculture within ten years, for reasons of animal welfare, environmental despoliation, human and animal health, and social justice. Most dramatically, I was able to broker an agreement between the Humane Society of the United States and the Colorado Livestock Association passing a jointly sponsored farm animal welfare law in Colorado in 2008, abolishing sow stalls and veal crates.

The agriculture community in the U.S. has been far behind societal concern. There is one monumental conceptual error that is omnipresent in the agricultural industry's discussions of animal welfare—an error of such magnitude that it trivializes the industry's responses to ever-increasing societal concerns about the treatment of agricultural animals. When one discusses farm animal welfare with industry groups or with the American Veterinary Medical Association, one finds the same response—animal welfare is solely a matter of “sound science”.

Those of us serving on the Pew Commission, better known as the National Commission on Industrial Farm Animal Production, encountered this response regularly during our dealings with industry representatives. This commission studied intensive animal agriculture in the U.S. [13]. For example, one representative of the Pork Producers, testifying before the Commission, answered that while people in her industry were quite “nervous” about the Commission, their anxiety would be allayed were we to base all of our conclusions and recommendations on “sound science”. Hoping to rectify the error in that comment, as well as educate the numerous industry representatives present, I responded to her as follows: “Madame, if we on the Commission were asking the question of how to raise swine in confinement, science could certainly answer that question for us. But that is not the question the Commission, or society, is asking. What we are asking is, ought we raise swine in confinement? And to this question, science is not relevant”. Judging by her “huh”, I assume I did not make my point.

Questions of animal welfare are at least partly “ought” questions, questions of ethical obligation. The concept of animal welfare is an ethical concept to which, once understood, science brings relevant data. When we ask about an animal's welfare, or about a person's welfare, we are asking about what we owe the animal, and to what extent. A document called the CAST report, first published by U.S. Agricultural scientists in the early 1980's, discussed animal welfare, it affirmed that the necessary and sufficient conditions for attributing positive welfare to an animal were represented by the animals' productivity. A productive animal enjoyed positive welfare; a non-productive animal enjoyed poor welfare [14].

This notion was fraught with many difficulties. First of all, productivity is an economic notion predicated of a whole operation; welfare is predicated of individual animals. An operation, such as caged laying hens may be quite profitable if the cages are severely over-crowded, yet the individual hens do not enjoy good welfare. Second, as we shall see, equating productivity and welfare is, to some significant extent, legitimate under husbandry conditions, where the producer does well if and only if the animals do well, and square pegs, as it were, are fitted into square holes with as little friction as possible (as when pigs live outside). Under industrial conditions, however, animals do not naturally fit in the niche or environment in which they are kept, and are subjected to “technological sanders” that allow for producers to force square pegs into round holes—antibiotics, feed additives, hormones, air handling systems—so the animals do not die and produce more and more kilograms of meat or milk. Without these technologies, the animals could not be productive. We will return to the contrast between husbandry and industrial approaches to animal agriculture.

The key point to recall here is that even if the CAST Report definition of animal welfare did not suffer from the difficulties we outlined, it is still an ethical concept. It essentially says “what we owe animals and to what extent is simply what it takes to get them to create profit”. This in turn would imply that the animals are well-off if they have only food, water, and shelter, something the industry has sometimes asserted. Even in the early 80's, however, there were animal advocates and others who would take a very different ethical stance on what we owe farm animals. Indeed, the famous five freedoms articulated in Britain by the Farm Animal Welfare Council during the 1970's (even before the CAST Report) represents quite a different ethical view of what we owe animals, when it affirms that:

The welfare of an animal includes its physical and mental state and we consider that good animal welfare implies both fitness and a sense of well-being. Any animal kept by man, must at least, be protected from unnecessary suffering.

We believe that an animal's welfare, whether on farm, in transit, at market or at a place of slaughter should be considered in terms of ‘**five freedoms**’ (see www.fawc.org.uk):

1. **Freedom from Hunger and Thirst**—by ready access to fresh water and a diet to maintain full health and vigor.
2. **Freedom from Discomfort**—by providing an appropriate environment including shelter and a comfortable resting area.
3. **Freedom from Pain, Injury or Disease**—by prevention or rapid diagnosis and treatment.
4. **Freedom to Express Normal Behavior**—by providing sufficient space, proper facilities and company of the animal's own kind.
5. **Freedom from Fear and Distress**—by ensuring conditions and treatment which avoid mental suffering.

Clearly, the two definitions contain very different notions of our moral obligation to animals (and there is an indefinite number of other definitions). Which is correct, of course, cannot be decided by gathering facts or doing experiments—indeed which ethical framework one adopts will in fact determine the shape of science studying animal welfare.

To clarify: suppose you hold the view that an animal is well-off when it is productive, as per the CAST Report. The role of your welfare science in this case will be to study what feed, bedding, temperature, *etc.* are most efficient at producing the most meat, milk, or eggs for the least money—much what animal and veterinary science does today. On the other hand, if you take the FAWC view of welfare, your efficiency will be constrained by the need to acknowledge the animal's natural behavior and mental state, and to assure that there is minimal pain, fear, distress and discomfort—not factors in the CAST view of welfare unless they have a negative impact on economic productivity. Thus, in a real sense, sound science does not determine your concept of welfare; rather, your concept of welfare determines what counts as sound science!

The failure to recognize the inescapable ethical component in the concept of animal welfare leads inexorably to those holding different ethical views talking past each other. Thus, producers ignore questions of animal pain, fear, distress, confinement, truncated mobility, bad air quality, social isolation, and impoverished environment unless any of these factors impact negatively on the “bottom line”. Animal advocates, on the other hand, give such factors primacy, and are totally unimpressed with how efficient or productive the system may be.

A major question obviously arises here. If the notion of animal welfare is inseparable from ethical components, and people's ethical stance on obligations to farm animals differ markedly across a highly diverse spectrum, whose ethic is to predominate and define, in law or regulation, what counts as “animal welfare”? It is to this issue we now turn.

What is the nature of the emerging new ethical thinking that underlies and informs the dramatic social changes just discussed? Although society has always had an articulated ethic regarding animal treatment, that ethic has been very minimalistic, leaving most of the issue of animal treatment to people's personal ethic, rather than to the social ethic. Since Biblical times, that limited social ethic has forbidden deliberate, willful, sadistic, deviant, purposeless, unnecessary infliction of pain and suffering on animals, or outrageous neglect, such as not feeding or watering. Beginning in the early nineteenth century, this set of prohibitions was articulated in the anti-cruelty statutes of the laws in all civilized societies [15]. But even in Biblical and medieval times, the social ethic inveighed against cruelty. The Old Testament injunctions against yoking an ox and an ass together to a plow, or muzzling the ox when it is being used to mill grain, or seething a calf in its mother's milk, all reflect concern with, and abhorrence for what the Rabbinical tradition called tsaar baalei chaiim; the suffering of living things. In the Middle Ages, St. Thomas Aquinas [16], while affirming that, lacking a soul, animals enjoyed no moral status, nonetheless strictly forbade cruelty, on the grounds that permitting such behavior towards animals would encourage its spreading to human beings, an insight buttressed by over two decades of recent research [17]. Numerous serial killers have evidenced early abusive behavior towards animals, as have many of the youths in the U.S. who in recent years wrought massacres on their peers.

For the overwhelming majority of human history, until some four decades ago, the anti-cruelty ethic served as the only socially articulated moral principle for animal treatment. Except for a few sporadic voices following in the wake of Darwin's articulation of human-animal continuity, no one spoke of animals' rights, nor did society have moral concepts for animal treatment that went “beyond cruelty.” The obvious question that presents itself is this: What has occurred during the last half century which led to social disaffection with the venerable ethic of anti-cruelty and to strengthening of the anti-cruelty laws, which now make cruelty a felony in almost 40 states.

In a study commissioned by USDA to answer this question, I distinguished a variety of social and conceptual reasons [18]:

(1) Changing demographics and consequent changes in the paradigm for animals:
  Whereas at the turn of the century, more than half the population was engaged in producing food for the rest, today only some 1.5% of the U.S. public is engaged in production agriculture[19]. One hundred years ago, if one were to ask a person in the street, urban or rural, to state the words that come into their mind when one says “animal”, the answer would doubtless have been “horse”, “cow”, “food”, “work”, *etc.* Today, however, for the majority of the population, the answer is “dog”, “cat”, “pet”. Repeated studies show that almost 100% of the pet-owning population views their animals as “members of the family” [20] and virtually no one views them as an income source. Divorce lawyers note that custody of the dog can be as thorny an issue as custody of the children!
(2) We have lived through a long period of ethical soul-searching
  For almost 50 years society has turned its “ethical searchlight” on humans traditionally ignored or even oppressed by the consensus ethic—blacks, women, the handicapped, other minorities. The same ethical imperative has focused attention on our treatment of the non-human world—the environment and animals. Many leaders of the activist animal movement in fact have roots in earlier movements—civil rights, feminism, homosexual rights, children's rights, labor.
(3) The media has discovered that “animals sell papers”
  One cannot channel-surf across normal television service without being bombarded with animal stories, real and fictional. (A *New York Times* reporter recently told me that more time on cable TV in New York City is devoted to animals than to any other subject.) Recall, for example, the extensive media coverage a decade ago of some whales trapped in an ice-floe, and freed by a Russian ice-breaker. This was hardly an overflowing of Russian compassion—an oxymoronic notion applied to a people who gave us pogroms, the Gulag, and Stalinism. Rather, someone in the Kremlin was bright enough to realize that liberating the whales was an extremely cheap way to score points with U.S. public opinion.
(4) Strong and visible arguments have been advanced in favor of raising the status of animals by philosophers, scientists and celebrities [21,22,23,24,25,26].
(5) Changes in the nature of animal use demanded new moral categories
  In my view, while all of the reasons listed above are relevant, they are nowhere nearly as important as the precipitous and dramatic changes in animal use that occurred after World War II. These changes were, first of all, huge conceptual changes in the nature of agriculture and second the rise of significant amounts of animal research and testing.

For virtually all of human history, animal agriculture was based foursquare in animal husbandry. Husbandry, derived from the old Norse word “hus/band,” bonded to the household, meant taking great pains to put one's animals into the best possible environment one could find to meet their physical and psychological natures which, following Aristotle, I call telos [23], and then augmenting their ability to survive and thrive by providing them with food during famine, protection from predation, water during drought, medical attention, help in birthing, and so on. Thus traditional agriculture was roughly a fair contract between humans and animals, with both sides being better off in virtue of the relationship. Husbandry agriculture was about putting square pegs into square holes, round pegs into round holes, and creating as little friction as possible doing so. So powerful is the notion of husbandry, in fact, that when the Psalmist seeks a metaphor for God's ideal relationship to humans, he seizes upon the shepherd in the 23rd Psalm:

The Lord is my shepherd; I shall not want; He maketh me to lie down in green pastures; He leadeth me beside still waters; He restoreth my soul.

We wish no more from God than what the husbandman provides for his sheep. In husbandry, a producer did well if and only if the animals did well, so productivity was tied to welfare. No social ethic was thus needed to ensure proper animal treatment; only the anti-cruelty designed to deal with sadists and psychopaths was needed to augment husbandry. Self-interest virtually assured good treatment.

After World War II, this beautiful contract was broken by humans. Symbolically, at universities, Departments of Animal Husbandry became Departments of Animal Science, defined not as care, but as “the application of industrial methods to the production of animals” to increase efficiency and productivity. With “technological sanders”—hormones, vaccines, antibiotics, air-handling systems, mechanization—we could force square pegs into round holes, and place animals into environments where they suffered in ways irrelevant to productivity. If a nineteenth century agriculturalist had tried to put 100,000 egg-laying hens in cages in a building, they all would have died of disease in a month; today such systems dominate.

The new approach to animal agriculture was not the result of cruelty, bad character or even insensitivity. It developed rather out of perfectly decent, prima facie plausible motives that were a product of dramatic significant historical and social upheavals that occurred after World War II. At that point in time, agricultural scientists and government officials became extremely concerned about supplying the public with cheap and plentiful food for a variety of reasons. In the first place, after the Dust Bowl and the Great Depression, many people in the U.S. had soured on farming. Second, reasonable predictions of urban and suburban encroachment on agricultural land were being made, with a resultant diminution of land for food production. Third, many farm people had been sent to both foreign and domestic urban centers during the war, thereby creating a reluctance to return to rural areas that lacked excitement; recall the song of the 40's “How are you gonna keep ‘em down on the farm now that they’ve seen Paree?” Fourth, having experienced the spectre of literal starvation during the Great Depression, the American consumer was, for the first time in history, fearful of an insufficient food supply. Fifth, projection of major population increases further fueled concern.

When the above considerations of loss of land and diminution of agricultural labor are coupled with the rapid development of a variety of technological modalities relevant to agriculture during and after World War II and with the burgeoning belief in technologically-based economics of scale, it was probably inevitable that animal agriculture would become subject to industrialization. This was a major departure from traditional agriculture and a fundamental change in agricultural core values—industrial values of efficiency and productivity replaced and eclipsed the traditional values of “way of life” and husbandry.

There is thus no question that industrialized agriculture, including animal agriculture, is responsible for greatly increased productivity. It is equally clear that the husbandry associated with traditional agriculture has changed significantly as a result of industrialization. One of my colleagues, a cow-calf cattle specialist, says that the worst thing that ever happened to his department is betokened by the name change from Animal Husbandry to Animal Science. No husbandry person would ever dream of feeding sheep meal, poultry waste, or cement dust to cattle, but such “innovations” are entailed by an industrial/efficiency mind-set.

In addition, in the mid-twentieth century there arose large scale use of animals in research and testing for toxicity. This too was an unprecedented large-scale use of animals, lacking the fairness of husbandry agriculture.

A moment's reflection on the development of large-scale animal research and high-technology agriculture elucidates why these innovations have led to the demand for a new ethic for animals in society. In a nutshell, these new developments represent a radically different playing field of animal use from the one that characterized most of human history; in the modern world of agriculture and animal research, the traditional anti-cruelty ethic grows increasingly less applicable. A thought experiment makes this clear. Imagine a pie chart that represents all the suffering that animals experience at human hands today. What percentage of that suffering is a result of intentional cruelty of the sort condemned by the anticruelty ethic and laws? When I ask my audiences this question—whether scientists, agriculturalists, animal advocates, or members of the general public—I always get the same response: only a fraction of 1 percent. Few people have ever witnessed overt, intentional cruelty, which is thankfully rare.

On the other hand, people realize that biomedical and other scientific research, toxicological safety testing, uses of animals in teaching, pharmaceutical product extraction from animals, and so on all produce far more suffering than does overt cruelty. This suffering comes from creating disease, burns, trauma, fractures, and the like in animals in order to study them; producing pain, fear, learned helplessness, aggression, and other states for research; poisoning animals to study toxicity; and performing surgery on animals to develop new operative procedures. In addition, suffering is engendered by the housing of research animals. Indeed, a prominent member of the biomedical research community has argued that the discomfort and suffering that animals used in research experience by virtue of being housed under conditions that are convenient for us, but inimical to their biological natures—for example, keeping rodents, which are nocturnal, burrowing creatures, in polycarbonate crates under artificial, full-time light—far exceed the suffering produced by invasive research protocols [27].

Now it is clear that farmers and researchers are not intentionally cruel—they are motivated by plausible and decent intentions: to cure disease, advance knowledge, ensure product safety, provide cheap and plentiful food. Nonetheless, they may inflict great amounts of suffering on the animals they use. Furthermore, the traditional ethic of anti-cruelty and the laws expressing it had no vocabulary for labeling such suffering, since researchers were not maliciously intending to hurt the animals. Indeed, this is eloquently marked by the fact that the cruelty laws exempt animal use in science and standard agricultural practices from their purview. Therefore, a new set of concepts beyond cruelty and kindness was needed to discuss the issues associated with burgeoning research animal use and industrial agriculture.

Society eventually became aware that new kinds of suffering were engendered by modern agriculture. Once again, producers could not be categorized as cruel, yet they were responsible for new types of animal suffering on at least four fronts:

1. Production diseases arise from the new ways the animals are produced. For example, liver abscesses in cattle are a function of certain animals' responses to the high-concentrate, low-roughage diet that characterizes feedlot production. (That is, of course, not the only cause of liver abscesses.) Although a certain percentage of the animals get sick and die, the overall economic efficiency of feedlots is maximized by the provision of such a diet. The ideas of a method of production creating diseases that were “acceptable” would be anathema to a husbandry agriculturalist.
2. The huge scale of industrialized agricultural operations and the small profit margin per animal militate against the sort of individual attention that typified much of traditional agriculture. In traditional dairies 50 years ago, one could make a living with a herd of 50 cows. Today, one needs literally thousands. In the U.S., dairies may have 10,000 cows.
3. Another new source of suffering in industrialized agriculture results from physical and psychological deprivation for animals in confinement: lack of space, lack of companionship for social animals, inability to move freely, boredom, austerity of environments, and so on. Since the animals evolved for adaptation to extensive environments but are now placed in truncated environments, such deprivation is inevitable. This was not a problem in traditional, extensive agriculture.
4. In confinement systems, workers may not be “animal smart”; the “intelligence,” such as it is, is in the mechanized system. Instead of husbandmen, workers in swine factories are minimum wage, often animal-ignorant labor. So there is often no empathy with, or concern for, the animals.

These sources of suffering, like the ones in research, are again not captured by the vocabulary of cruelty, nor are they proscribed or even acknowledged by the laws based on the anti-cruelty ethic. Furthermore, they typically do not arise under traditional agriculture and its ethic of husbandry.

A few years ago, I experienced some sharply contracting incidents which dramatically highlight the moral difference between intensive and extensive agriculture. That particular year, Colorado cattle ranchers, paradigmatic exemplars of husbandry, were afflicted by a significant amount of scours. Over two months, I talked to a half dozen rancher friends of mine. Every single one had experienced trouble with scours, and every one had spent more on treating the disease than was economically justified by the calves' monetary value. When I asked these men why they were being what an economist would term “economically irrational,” they were quite adamant in their response: “It's part of my bargain with the animal; part of caring for them,” one of them said.

It is, of course, the same ethical outlook that leads ranch wives to sit up all night with sick marginal calves, sometimes for days in a row. If the issues were strictly economic, these people would hardly be valuing their time at 50 ¢ per hour—including their sleep time!

Now in contrast to these uplifting moral attitudes, consider the following: One of my animal scientist colleagues related to me that his son-in-law was an employee in a large, total confinement swine operation. As a young man he had raised and shown pigs, keeping them semi-extensively. One day he detected a disease among the feeder pigs in the confinement facility where he works, which necessitated killing them with a blow to the head, since this operation did not treat individual animals, their profit margin being allegedly too low. Out of his long established husbandry ethic, he came in on his own time with his own medicine to treat the animals. He cured them! Management's response was to fire him on the spot for violating company policy! He kept his job and escaped with a reprimand only when he was able to prove that he had expended his own—not the company's—resources. He continued to work for them, but felt that his health has suffered in virtue of what I have called the “moral stress” he experiences every day; the stress growing out of the conflict between what he is told to do and how he morally believes he should be treating the animals. Eventually, he left agriculture altogether. The above-detailed contrasting incidents, better than anything else I know, eloquently illustrate the large gap between the ethics of husbandry and industry. (Many confinement operations are run by accountants, not by animal science or animal husbandry people.)

Given that the old anti-cruelty ethic did not apply to animal research or confinement agriculture, society needed new ethical concepts to express its concern about these new uses. But ethical concepts do not arise *ex nihilo*.

Plato taught us a very valuable lesson about effecting ethical change. If one wishes to change another person's—or society's—ethical beliefs, it is much better to remind than to teach or, in my martial arts metaphor, to use judo rather than sumo. In other words, if you and I disagree ethically on some matter, it is far better for me to show you that what I am trying to convince you of is already implicit—albeit unnoticed—in what you already believe. Similarly, we cannot force others to believe as we do (sumo); we can, however, show them that their own assumptions, if thought through, lead to a conclusion different from what they currently entertain (judo). These points are well-exemplified in 20th century U.S. history. Prohibition was sumo, not judo—an attempt to forcefully impose a new ethic about drinking on the majority by the minority. As such, it was doomed to fail, and in fact people drank more during Prohibition. Contrast this with Lyndon Johnson's civil rights legislation. As himself a Southerner, Johnson realized that even Southerners would acquiesce to the following two propositions:

1. All humans should be treated equally, and black people were human—they just had never bothered to draw the relevant conclusion.
2. If Johnson had been wrong about this point, if “writing this large” in the law had not “reminded” people, civil rights would have been as ineffective as Prohibition!

So society was faced with the need for new moral categories and laws that reflect those categories in order to deal with animal use in science and agriculture and to limit the animal suffering with which it is increasingly concerned. At the same time, recall that western society has one through almost fifty years of extending its moral categories for *humans* to people who were morally ignored or invisible—women, minorities, the handicapped, children, citizens of the third world. As we noted earlier, new and viable ethics do not emerge *ex nihilo*. So a plausible and obvious move is for society to continue in its tendency and *attempt to extend the moral machinery it has developed for dealing with people, appropriately modified, to animals.* And this is precisely what has occurred. Society has taken elements of the moral categories it uses for assessing the treatment of people and is in the process of modifying these concepts to make them appropriate for dealing with new issues in the treatment of animals, especially their use in science and confinement agriculture.

What aspect of our ethic for people is being so extended? One that is, in fact, quite applicable to animal use, is the fundamental problem of weighing the interests of the individual against those of the general welfare. Different societies have provided different answers to this problem. Totalitarian societies opt to devote little concern to the individual, favoring instead the state, or whatever their version of the general welfare may be. At the other extreme, anarchical groups such as communes give primacy to the individual and very little concern to the group—hence they tend to enjoy only transient existence. In our society, however, a balance is struck. Although most of our decisions are made to the benefit of the general welfare, fences are built around individuals to protect their fundamental interests from being sacrificed to the majority. Thus we protect individuals from being silenced even if the majority disapproves of what they say; we protect individuals from having their property seized without recompense even if such seizure benefits the general welfare; we protect individuals from torture even if they have planted a bomb in an elementary school and refuse to divulge its location. We protect those interests of the individual that we consider essential to being human, to *human nature*, from being submerged, even by the common good. Those moral/legal fences that so protect the individual human are called *rights* and are based on plausible assumptions regarding what is essential to being human.

It is this notion to which society in general is looking in order to generate the new moral notions necessary to talk about the treatment of animals in today's world, where cruelty is not the major problem but where such laudable, general human welfare goals as efficiency, productivity, knowledge, medical progress, and product safety are responsible for the vast majority of animal suffering. People in society are seeking to “build fences” around animals to protect the animals and their interests and natures from being totally submerged for the sake of the general welfare, and are trying to accomplish this goal by going to the legislature. In husbandry, this occurred automatically; in industrialized agriculture, where it is no longer automatic, people wish to see it legislated.

It is necessary to stress here certain things that this ethic, in its mainstream version, is *not* and does not attempt to be. As a mainstream movement, it does not try to give human rights to animals. Since animals do not have the same natures and interests flowing from these natures as humans do, human rights do not fit animals. Animals do not have basic natures that demand speech, religion, or property; thus according them these rights would be absurd. On the other hand, animals have natures of their own and interests that flow from these natures, and the thwarting of these interests matters to animals as much as the thwarting of speech matters to humans. The agenda is not, for mainstream society, making animals have the same rights as people. It is rather preserving the common-sense insight that “fish gotta swim and birds gotta fly,” and suffer if they don't.

This new ethic is conservative, not radical, harking back to the animal use that necessitated and thus entailed respect for the animals' natures. It is based on the insight that what we do to animals *matters* to them, just as what we do to humans matters to them, and that consequently we should respect that mattering in our treatment of use of animals as we do in our treatment and use of humans. *And since respect for animal nature is no longer automatic as it was in traditional husbandry agriculture, society is demanding that it be encoded in law*. Significantly, in 2004, no fewer than 2,100 bills pertaining to animal welfare were proposed in U.S. state legislatures. 90+ law schools now teach animal law.

With regards to animal agriculture, the pastoral images of animals grazing on pasture and moving freely are iconic. As the 23rd Psalm indicates, people who consume animals wish to see the animals live decent lives, not lives of pain, distress and frustration. It is for this reason in part that industrial agriculture conceals the reality of its practices from a naïve public—witness Perdue's advertisements about raising “happy chickens”, or the California “happy cow” ads. As ordinary people discover the truth, they are shocked. When I served on the Pew Commission and other commissioners had their first view of sow stalls, many were in tears and all were outraged.

Just as our use of people is constrained by respect for the basic elements of human nature, people wish to see a similar notion applied to animals. Animals, too, have natures, what I call telos following Aristotle—the “pigness of the pig”, the “cowness of a cow”. Pigs are “designed” to move about on soft loam, not to be in gestation crates. If this no longer occurs naturally, as it did in husbandry, people wish to see it legislated. This is the mainstream sense of “animal rights”.

As property, strictly speaking, animals cannot have legal rights. But a functional equivalent to rights can be achieved by limiting human property rights. When I and others drafted the U.S. federal laws for laboratory animals, we did not deny that research animals were the property of researchers. We merely placed limits on their use of their property. I may own my car, but that does not mean I can drive it on the sidewalk or at any speed I choose. Similarly, our law states that if one hurts an animal in research, one must control pain and distress. Thus research animals can be said to have the right to have their pain controlled. [27]

In the case of farm animals, people wish to see their basic needs and nature, teloi, respected in the systems that they are raised. Since this no longer occurs naturally as it did in husbandry, it must be imposed by legislation or regulation. A Gallup poll conducted in 2003 shows that 75% of the public wants legislated guarantees of farm animal welfare [28]. This is what I call “animal rights as a mainstream phenomenon”. Legal codification of rules of animal care respecting animal telos is thus the form animal welfare takes where husbandry has been abandoned.

Thus, in today's world, the ethical component of animal welfare prescribes that the way we raise and use animals must embody respect and provision for their psychological needs and natures. It is therefore essential that industrial agriculture phase out those systems which cause animal suffering by violating animals' natures and replace them with systems respecting their natures.
